# Lateralizing Lung Logjam: A Unique Case of Concomitant Lateralizing Lung Abscess, Parapneumonic Effusion, and Pneumothorax

**DOI:** 10.7759/cureus.93410

**Published:** 2025-09-28

**Authors:** Parth Desai, Julie Gaudin, Sneh Parekh, Sai R Vulasala, Pramod Reddy

**Affiliations:** 1 Internal Medicine, University of Florida College of Medicine – Jacksonville, Jacksonville, USA; 2 Radiology, University of Florida College of Medicine – Jacksonville, Jacksonville, USA

**Keywords:** alcohol use disorder, aspiration pneumonitis, bronchopleural fistula, chest computed tomography, light's criteria, lung abscess, parapneumonic effusion, pneumothorax

## Abstract

The management of lung abscesses is well documented in medical literature; however, the concomitant presentation of a lung abscess with associated pneumothorax and parapneumonic effusion, within the same laterality of the lungs, is rare, with limited data in the literature regarding the management of such presentations. Here, we present the case and management of a 67-year-old male with this unique presentation. The management of which involved the separation of the individual entities with individualized treatment based upon the level of involvement within the lung parenchyma. Additionally, employing a multidisciplinary approach involving interventional radiology, pulmonology, and internal medicine was essential in diagnosis and management.

## Introduction

Lung abscesses are cavitary lesions composed of pus and necrotic tissue, often occurring secondary to aspiration [1]. Common risk factors include alcohol use disorder, underlying structural lung disease, and poor oral hygiene [1]. The mainstay of management includes empiric intravenous (IV) antibiotic coverage of anaerobic microorganisms, as lung abscesses secondary to aspiration commonly include organisms that inhabit the upper airway, particularly the oropharynx [2]. In most cases, antibiotics can be de-escalated to an oral regimen following clinical improvement without the need for further invention. If left untreated, lung abscesses may present a greater challenge and result in the development of parapneumonic effusions.

Parapneumonic effusions are exudative pleural fluid collections that occur as complications secondary to pneumonia or lung abscesses [3]. Parapneumonic effusions can be categorized on a spectrum, including uncomplicated and complicated effusions, and empyemas [3]. Uncomplicated parapneumonic effusions are sterile and typically resolve with antibiotic treatment of the underlying predisposing pneumonia or abscess. Complicated parapneumonic effusions involve signs of infection and bacteria within the pleural fluid, and require percutaneous drainage in addition to antibiotics [3]. Without proper treatment, a parapneumonic effusion may progress to an empyema, which is characterized by aspiration of pus and ultimately requires drainage [3]. Furthermore, while early empiric antibiotic therapy is indicated in all parapneumonic effusions, the decision for drainage is based on the anatomical characteristics, bacteriology, and chemistry of the pleural fluid. [4,5].

Spontaneous pneumothoraces are characterized by air within the pleural cavity and can be described as primary or secondary. In contrast to primary pneumothoraces, secondary pneumothoraces are associated with underlying disease [6]. Management of spontaneous pneumothoraces depends on the size and presentation. Smaller, asymptomatic cases may be observed; however, larger pneumothoraces or recurrence of pneumothoraces may require needle aspiration, pigtail catheter placement, tube thoracostomy, pleurodesis, or surgical intervention [7]. However, management of pneumothoraces in the setting of lung abscesses or parapneumonic pleural effusions presents a unique challenge that is not widely described within the current literature [8].

Each of these pathologies inherently presents its own clinical difficulties, and when combined, can present a challenging clinical presentation and management dilemma as to which pathologies to approach first and in which fashion. Here, we present the case of a 67-year-old male who presented with a lung abscess with progression to parapneumonic effusion and spontaneous pneumothorax, along with a discussion on management guidelines.

## Case presentation

A 67-year-old male with a past medical history of alcohol use disorder, consuming approximately 12 beers daily, alcoholic cirrhosis, tobacco use disorder with a 40-pack-year history of smoking, COPD, and essential hypertension, presented with complaints of hemoptysis with clots and shortness of breath for the past three days. The patient further endorsed subjective fevers, which began seven days ago and self-resolved prior to the onset of his hemoptysis and shortness of breath. On presentation, he was tachycardic, tachypneic, hypertensive, hypoxemic, requiring two L of supplemental oxygen via nasal cannula, and had a low-grade fever. His physical exam was notable for diminished breath sounds on the right and wheezing on the left. Given that he met sepsis criteria with tachycardia, tachypnea, and low-grade fevers, he was started on empiric antibiotic coverage with vancomycin and piperacillin-tazobactam. The patient’s initial laboratory studies were notable for alkalosis, with respiratory compensation, and a neutrophil-predominant leukocytosis, as seen in Table 1.

**Table 1:** Initial laboratory values on admission.

Laboratory Tests	Patient Laboratory Values	Reference Range Values	Value Interpretation
Venous pH	7.49	7.34 - 7.36	Elevated
Venous pCO_2_ (mmHg)	46	35 - 45	Elevated
Venous pO_2_ (mmHg)	43	30 - 55	Normal
White Blood Cell Count (cells/L)	13.05 × 10^9^	4.5 - 11 × 10^9^	Elevated
Hemoglobin (grams/dL)	15.7	14.0 - 18.0	Normal
Neutrophil Percentage (%)	92	34 - 73	Elevated

**Figure 1:**
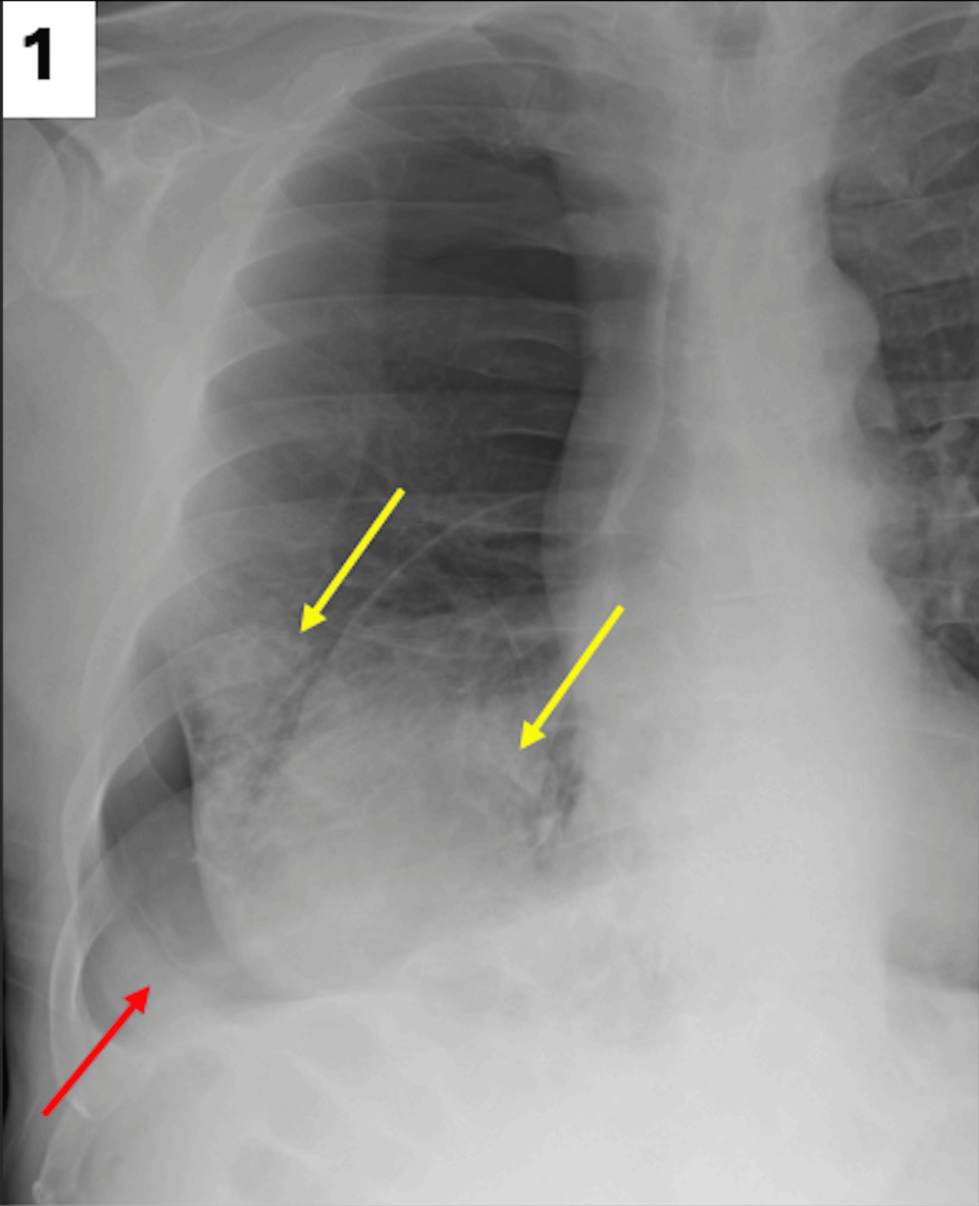
Initial chest X-ray obtained in the emergency department demonstrating radiolucency with absent lung markings along the right lung base, indicating pneumothorax (red arrow). Additionally, there are dense pulmonary opacities in the right lung base (yellow arrows), partially obscuring the right heart border.

Initially, a chest X-ray was obtained on admission, which demonstrated a large right-sided pneumothorax with near complete atelectasis of the right lung (Figure 1).

**Figure 2:**
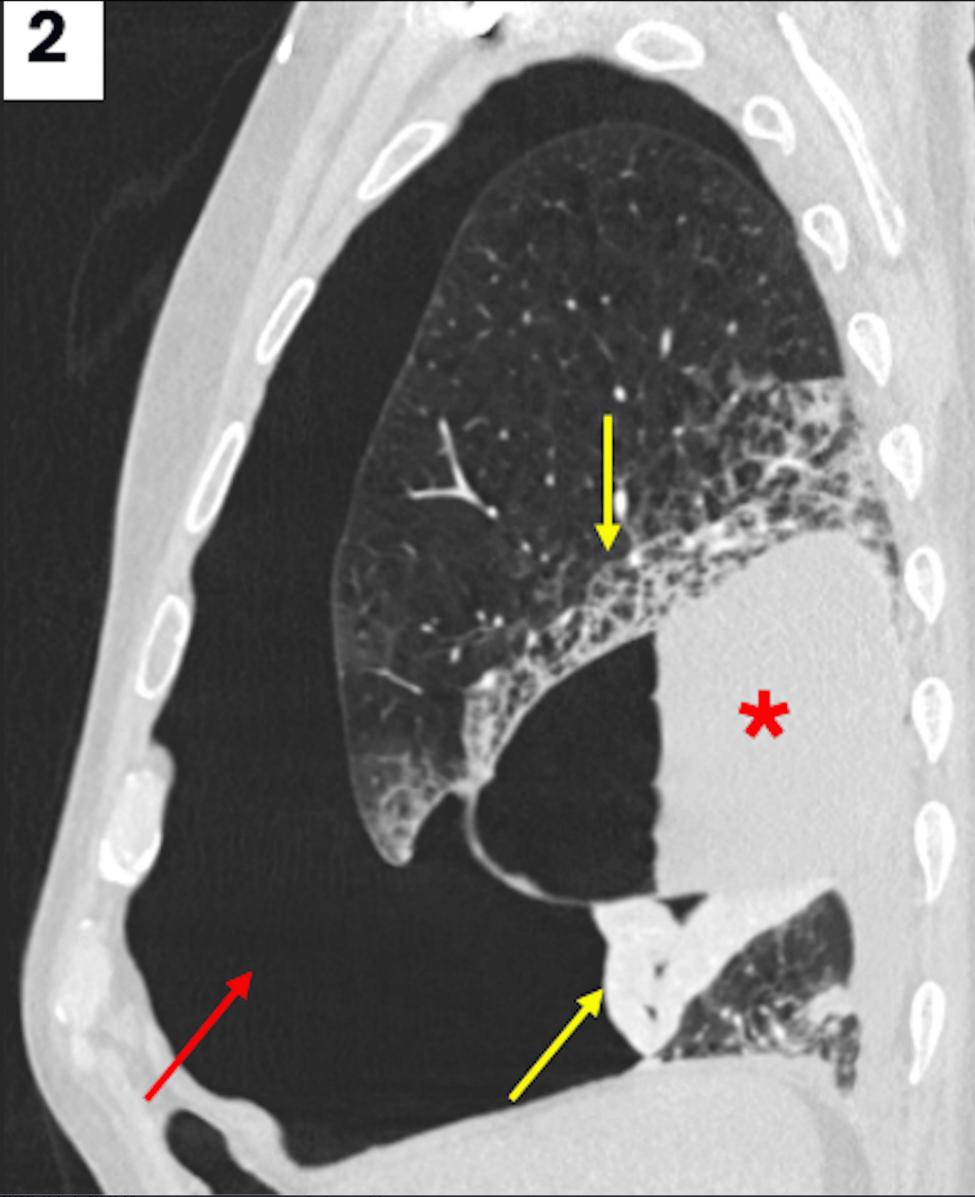
Sagittal CTA of the chest with PE protocol showing intravenous contrast in lung window, which demonstrates a moderate right pneumothorax (red arrow). There is a collection with air-fluid levels (red asterisk) along the right minor fissure, concerning for hydropneumothorax versus pyopneumothorax. There is also a collapse of the right middle lung lobe with interstitial opacities in the right upper lobe of the lung (yellow arrows). CTA: computed tomography angiography; PE: pulmonary embolism

A computed tomography angiography (CTA) of the chest, with pulmonary embolism (PE) protocol, was then ordered in the setting of the patient’s tachycardia, tachypnea, dyspnea, and hypoxemia with new oxygen requirements. The CTA provided further characterization of the right-sided pneumothorax, with the presence of right-sided air-fluid levels and collapse of the right lung (Figure 2).

**Figure 3:**
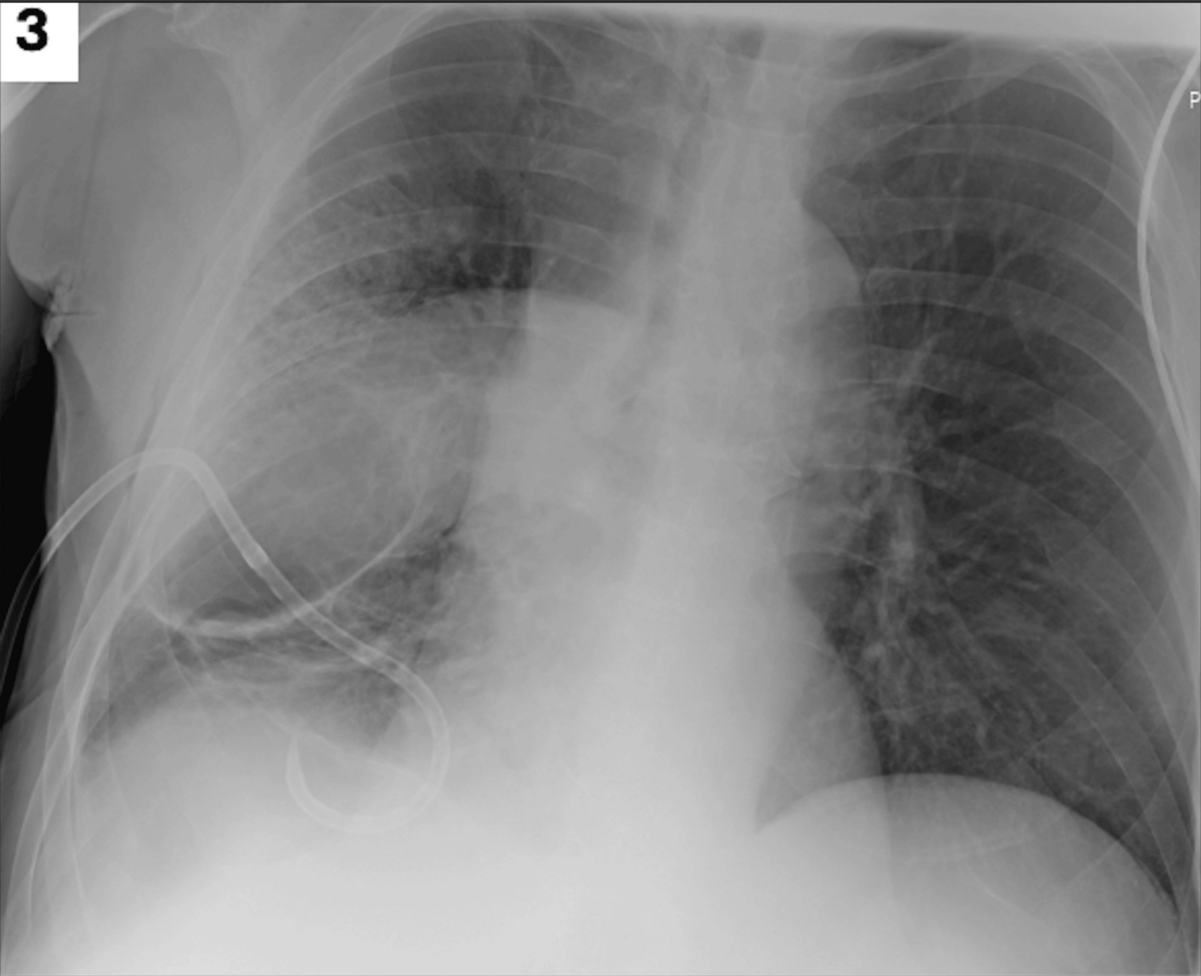
Repeat chest X-ray, post chest tube placement.

Thereafter, a 12 French pigtail catheter chest tube connected to suction was placed, with brown fluid drained. A repeat chest X-ray was obtained, post chest tube placement, still with the presence of the right-sided radiolucency, with mildly noted improvement at the right lung base (Figure 3).

Once the catheter chest tube was placed, fluid studies were obtained. The fluid was neutrophil-predominant, with significantly elevated pleural fluid protein, lactate dehydrogenase (LDH), and pH value. These values were compared to the patient’s serum total protein and serum LDH, as seen in Table 2.

**Table 2:** Pleural fluid studies from chest tube drainage and relevant corresponding serum studies. LDH: lactate dehydrogenase

Laboratory Tests	Patient Laboratory Values	Reference Range Values	Value Interpretation
Pleural Fluid Color	Brown	Straw-Colored	Abnormal
Pleural Fluid Neutrophils (%)	63	0 - 5	Elevated
Pleural Fluid Glucose (mg/dL)	119	60 - 100	Elevated
Pleural Fluid LDH (U/L)	846	Less than 50% of serum LDH value	Elevated
Pleural Fluid Protein (g/dL)	3.8	< 1.5	Elevated
Pleural Fluid pH	8.00	7.60 - 7.66	Elevated
Serum Total Protein (g/dL)	6.9	6.5 - 8.3	Elevated
Serum LDH (IU/L)	205	126 - 266	Normal

Given that the pleural fluid protein to serum total protein ratio was greater than 0.5 ( = 0.55), and his pleural fluid LDH to serum LDH ratio was greater than 0.6 ( = 4.13), per Light’s Criteria, fluid studies were consistent with an exudative pleural effusion. Furthermore, the patient had evidence of loculated fluid on the CTA PE protocol imaging, along with pleural fluid LDH level greater than three times the upper limit of normal for serum, also confirming the presence of a parapneumonic effusion.

Due to the initial concerns for sepsis with a now-identified source being the loculated air-fluid collection, concerning for a lung abscess, the patient was continued on broad-spectrum antibiotic coverage. Antibiotic coverage was expanded from vancomycin and piperacillin-tazobactam to vancomycin, cefepime, and metronidazole, for the increased lung penetration abilities that cefepime would provide. Pulmonology was initially consulted for drainage of the fluid collection; however, there was concern for intra-parenchymal involvement of the abscess, which could result in the possible creation of a bronchopleural fistula if it were to be prematurely drained.

**Figure 4:**
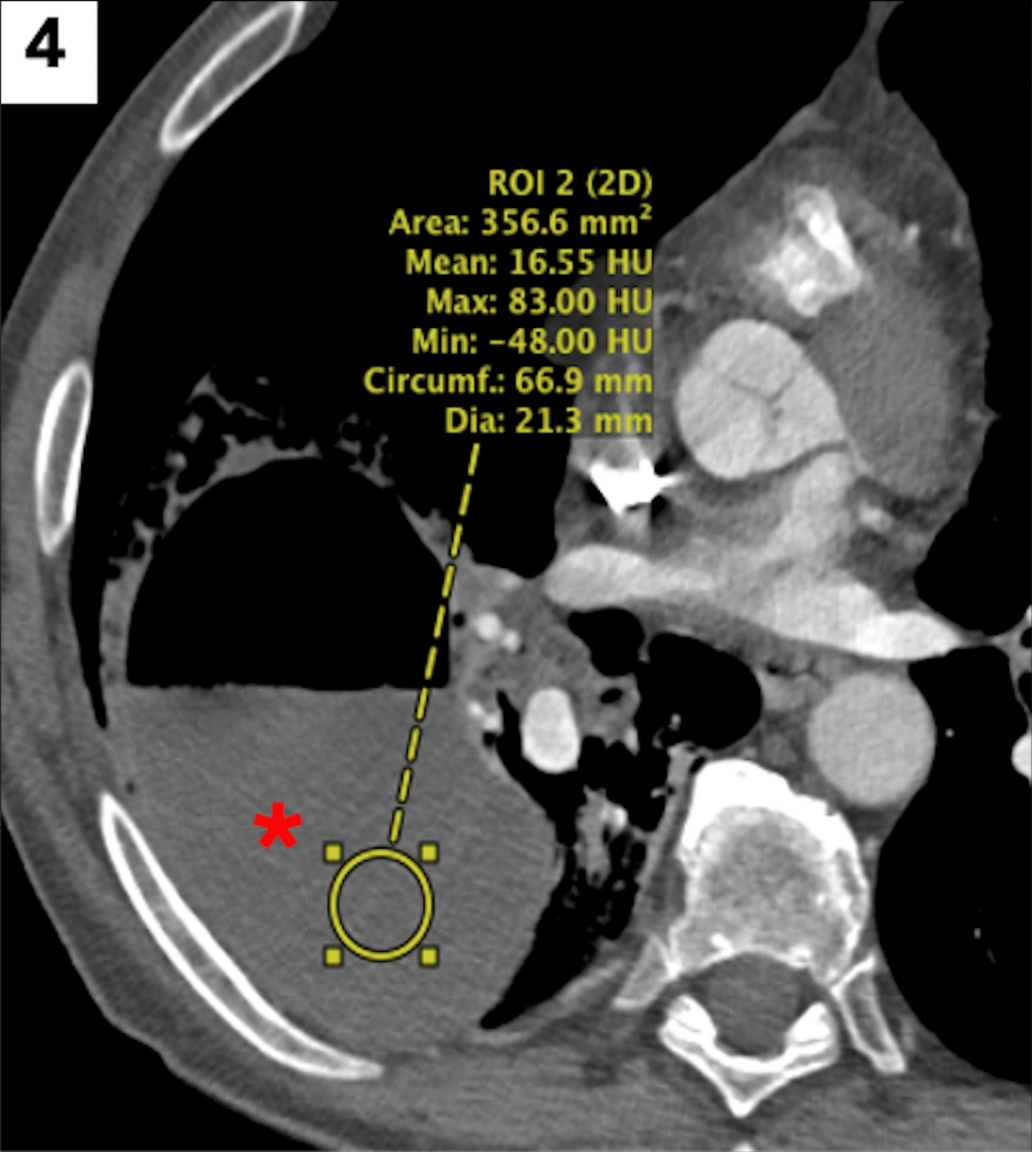
Axial CT of the chest in the soft tissue window, confirming the fluid attenuation of the collection with air-fluid levels (red asterisk). CT: computed tomography

**Figure 5:**
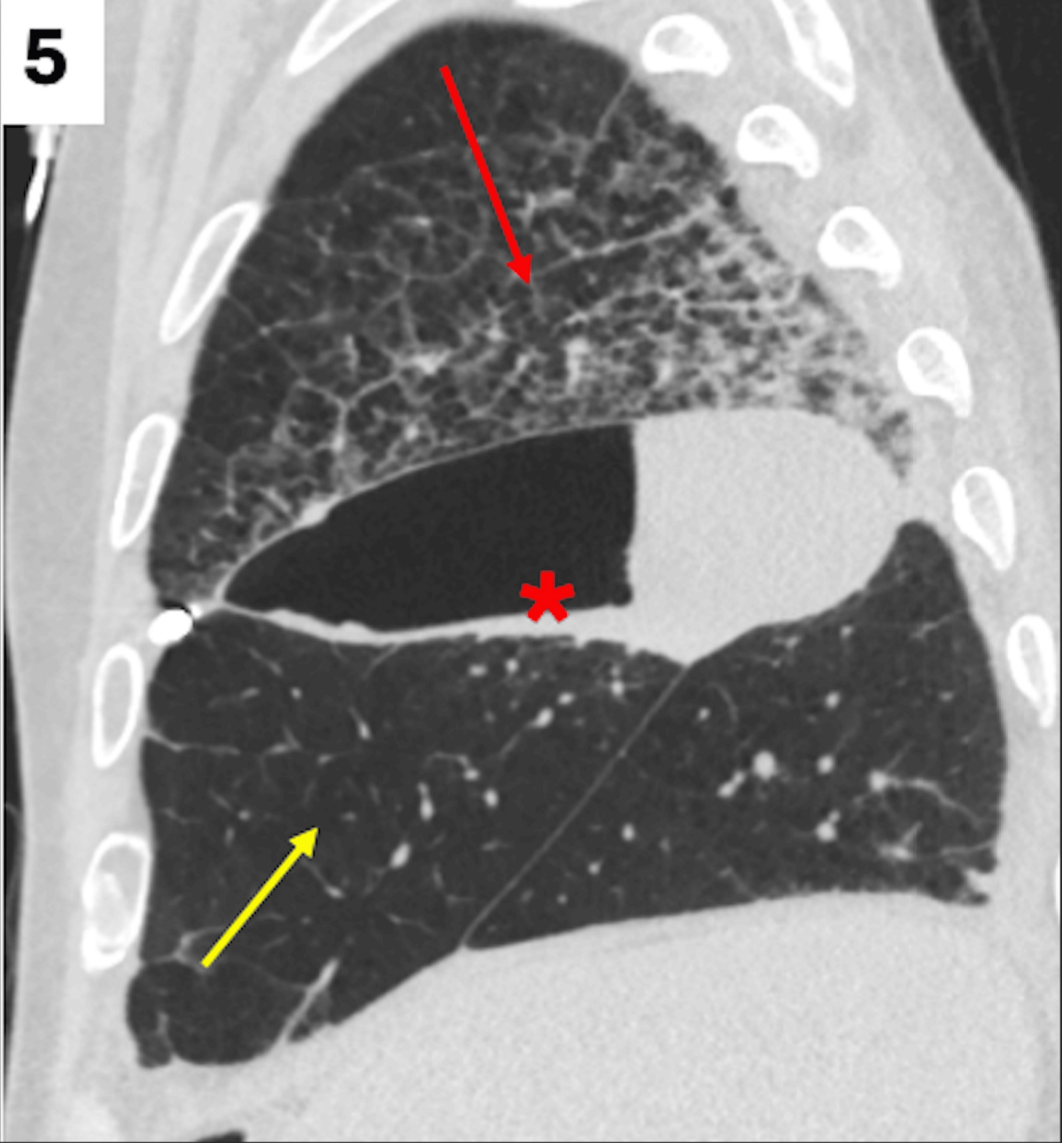
CT of the chest confirming resolution of the pneumothorax with re-expansion of the right middle lung lobe (yellow arrow), post chest tube placement. Interval worsening of the interstitial opacities in the right upper lung lobe is seen, which can be due to re-expansion pulmonary edema (red arrow). There is also an unchanged collection along the right minor fissure with thickening of the pleural layers (red asterisk), raising concern for a pyopneumothorax (red asterisk). CT: computed tomography

Thereafter, on hospital day 2, given a negative methicillin-resistant Staphylococcus aureus (MRSA) nares test, vancomycin was discontinued, with the patient continued on cefepime and metronidazole. Finally, a dedicated computed tomography (CT) of the chest without contrast was obtained to further characterize the nature of the abscess, which demonstrated association with a fissure that would be amenable to drainage, with a low risk of fistula creation (Figures 4-5).

**Figure 6:**
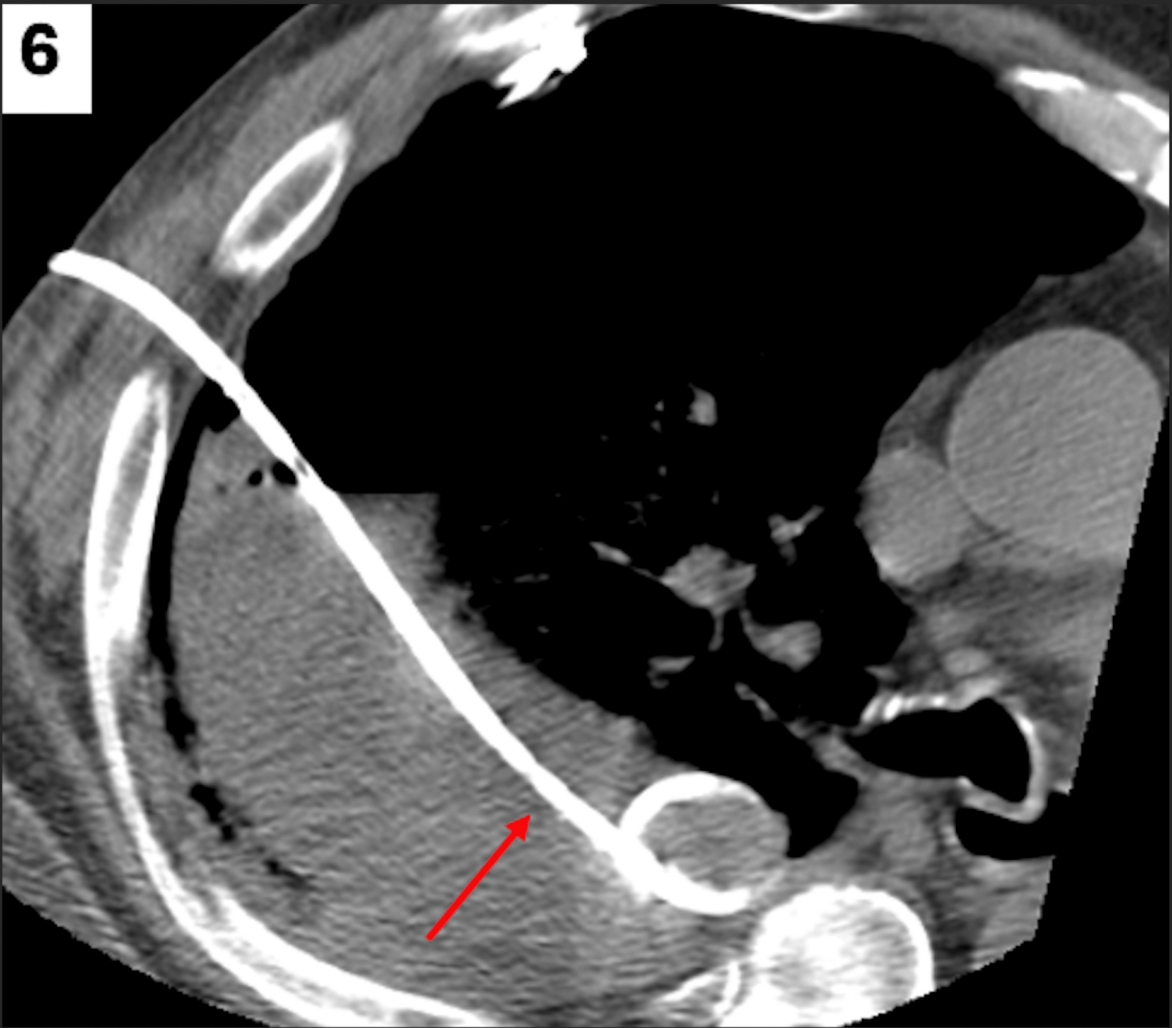
CT-guided pigtail drainage catheter placed into the pyopneumothorax for drainage (red arrow). CT: computed tomography

Interventional radiology was then consulted and placed a 10 French chest tube via CT guidance, with 218 mL of initial output drained within the first 24 hours (Figure 6).

**Figure 7:**
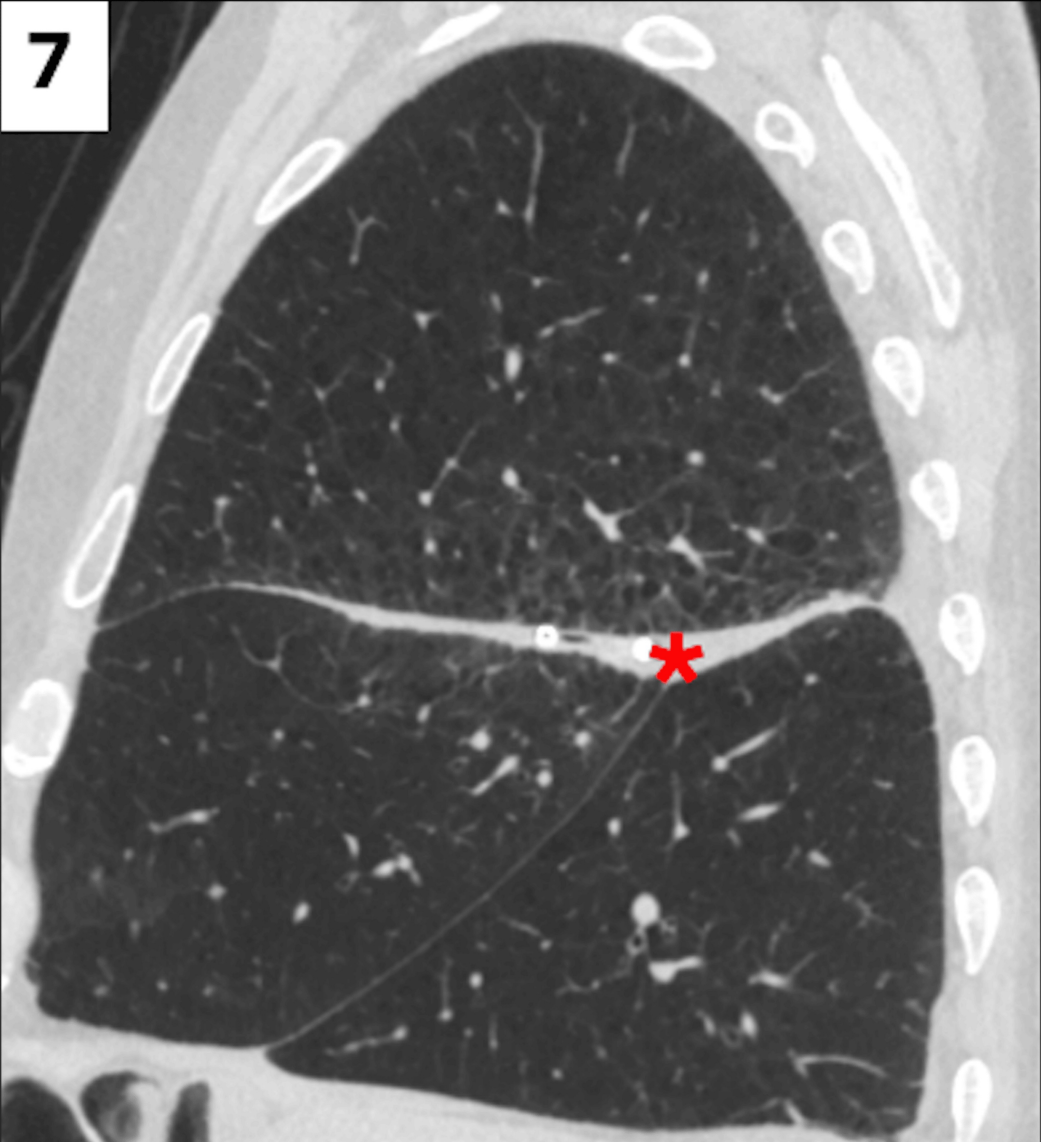
Follow-up CT of the chest in a sagittal view, demonstrating near complete resolution of the pyopneumothorax (red asterisk) and improvement in the previously identified right upper lobar interstitial pulmonary opacities. CT: computed tomography

Upon placement of the 10 French chest tube, the prior 14 French pigtail catheter was removed. Over the next five days, the patient continued to have drainage from the 10 French chest tube, and he remained hemodynamically stable and afebrile, prompting transition of antibiotics to an oral regimen of amoxicillin-clavulanate. After a total of seven days, on hospital day 10, the 10 French chest tube was removed with complete cessation of drainage output. A repeat CT of the chest was obtained upon removal of the chest tube, indicating near complete resolution of the abscess, parapneumonic effusion, and pneumothorax (Figure 7).

The patient was monitored for an additional 24 hours following removal of the chest tube, and remained stable after which he was discharged home on the oral amoxicillin-clavulanate regimen, which was continued for an additional four weeks.

## Discussion

Lung abscesses are cavitary lesions composed of pus and necrotic tissue [1]. Lung abscesses tend to occur due to aspiration and can occur in normal lung tissue in about 60% of cases [9,10]. Risk factors include conditions that impair consciousness or increase aspiration risk, along with structural lung disease, poor dental hygiene, and conditions affecting immune defense mechanisms [1,11]. Alcohol use disorder, a medical condition seen in our patient, promotes aspiration risk and immunosuppression via impairment of the mucociliary apparatus within the respiratory tract, predisposing to infection and delayed recovery [12,13]. Signs of a productive cough with purulent sputum or hemoptysis, also present in our patient, may indicate communication between the abscess and bronchus, posing greater challenges in disease management and an increased risk of complications [11].

Most lung abscesses are polymicrobial, involving anaerobic microorganisms found in oropharyngeal mucosa; however, up to 50% of cases are culture negative [9]. The mainstay of management includes empiric IV antibiotic coverage of anaerobic microorganisms [2]. De-escalation to an oral antibiotic regimen, such as amoxicillin-clavulanate as seen in our case, can be considered once a patient is afebrile, hemodynamically stable, and tolerating an oral diet [2]. Approximately 80-90% of cases respond to antibiotics without the need for percutaneous drainage [9,10]. However, there is now increasing evidence supporting early abscess drainage to accelerate recovery and reduce hospital length of stay [14]. In one meta-analysis, percutaneous drainage was found to have increased efficacy in the treatment of lung abscesses, reducing the length of hospital stay, when compared to conservative treatment with IV antibiotics alone [15]. However, percutaneous drainage imposes further risks of complications, including pneumothorax, hemothorax, infection of the pleural space, and formation of a bronchopleural fistula, which may necessitate surgical intervention [9]. Although most lung abscesses respond to antibiotic therapy alone, it is necessary to maintain high clinical suspicion for worsening infectious processes, including the development of complicated parapneumonic effusions and progression to pleural empyema, as was necessitated in our patient case.

Parapneumonic effusions are exudative pleural fluid collections and may occur as complications of pneumonia or a lung abscess [3,4]. In one retrospective analysis of 259 cases of pleural empyema, lower respiratory tract infections were found to be the leading cause in 93% of cases. Twenty-two of these cases (8.5%) also had lung abscesses, resulting in significantly higher risks of mortality [10]. Parapneumonic effusions are categorized as a progression from uncomplicated to complicated and finally, empyema [3]. The pathophysiology is further described in three stages, progressing from exudative to fibrinopurulent to an organized empyema involving fibroblast formation of a thick pleural peel, which prevents lung expansion [4,10,16]. These stages are characterized based on bacterial studies and pleural fluid analyses, including pleural pH, glucose, and lactate dehydrogenase. Pleural fluid pH less than 7.20 is the most sensitive measurement indicative of a complicated parapneumonic effusion [4]. CT chest imaging, as shown in this case, is often used to provide further characterization of parapneumonic effusions. Findings of fluid loculations, pleural thickening, and contrast enhancement secondary to fibrin deposition may change management.

Management of parapneumonic effusions includes early empiric antibiotics for all cases to prevent progression to an empyema. DNA amplification has been shown to isolate anaerobic organisms in 76% of pleural samples, necessitating empiric anaerobic coverage despite negative anaerobic cultures [5]. Duration of antibiotic therapy is based on clinical response and radiographic improvement, but complicated parapneumonic effusions require a longer duration of antibiotics, lasting at least three weeks [5]. Complicated parapneumonic effusions and empyemas additionally require drainage via percutaneous drainage or thoracostomy tube [17]. Progression to a pleural empyema despite effective antibiotics and drainage is seen in about 20% of cases of parapneumonic effusions [10]. Further complications that may require additional management include pleural thickening or fibrosis and the formation of a bronchopleural fistula [16]. Intrapleural fibrinolytics and surgical interventions, such as video-assisted thoracoscopic surgery (VATS), may be indicated in these cases of complications or in cases of treatment failure based on worsening clinical status or radiographic progression [5].

Proper diagnosis and management with close monitoring of clinical status are critical to improve outcomes in cases of parapneumonic effusions. In 2000, parapneumonic effusions were classified by the American College of Chest Physicians by anatomical fluid characteristics (size, presence or absence of loculations), bacteriology of pleural fluid (culture and Gram stain, presence of pus), and chemistry of pleural fluid (pleural fluid pH less than 7.20) [4,5]. From this classification, parapneumonic effusions are placed into one of four categories corresponding to an increased risk of poor outcomes in higher categories, in addition to treatment recommendations. Categories 1 and 2 can be treated using IV antibiotics without drainage. Categories 3 and 4 correspond to increased risk of poor outcomes, requiring drainage in addition to IV antibiotics [4,5].

In our presented case, the presence of a loculated effusion despite negative cultures, negative Gram stain, and pleural fluid pH greater than 7.20 describes a Category 3 complicated parapneumonic effusion, associated with a moderate risk of poor outcome and recommendation for drainage in addition to IV antibiotics. Initially, given the presence of the large right-sided pneumothorax, a pigtail catheter had to be placed to allow for small-volume drainage. This assisted in allowing space for lung re-expansion and improvement of the pneumothorax, while also providing fluid cultures to better characterize the associated parapneumonic effusion. Thereafter, percutaneous drainage was performed via a small-bore catheter for management of the complicated parapneumonic effusion and lung abscess. Drainage of the fluid, along with continued antibiotic administration, provided source control and symptomatic resolution. To our knowledge, no other reports exist in the current medical literature detailing concomitant, same-sided lung abscess, parapneumonic pleural effusion, and the presence of a pneumothorax. In a complicated lung abscess, bronchopleural fistula formation can occur with necrosis eroding the lung parenchyma. Given the concern for parenchymal involvement and formation of a bronchopleural fistula in our patient, repeat imaging was pursued, indicating a safe space for percutaneous drainage. In complicated lung abscesses, clinicians should maintain a high index of suspicion for repeat imaging prior to intervention due to the inherent risks present secondary to bronchopleural fistula formation. Overall, our case presents a unique challenge involving concurrent unilateral lung findings of an abscess with the development of a complicated parapneumonic effusion and large pneumothorax.

## Conclusions

Our case presented a unique presentation of a large pneumothorax in the setting of a complicated parapneumonic effusion with lung abscess formation; all confined to the right thorax. Our patient’s concomitant pathologies were likely predisposed secondary to recurrent aspiration with a known history of alcohol use disorder. The coinciding presentation of these unilateral findings is a rare occurrence with no specific incidence rates seen in current literature. This case details the unique challenge of the importance of sequential management and a multidisciplinary approach when such coinciding pathologies present.
